# Tobramycin and Amikacin Delay Adhesion and Microcolony Formation in *Pseudomonas aeruginosa* Cystic Fibrosis Isolates

**DOI:** 10.3389/fmicb.2017.01289

**Published:** 2017-07-11

**Authors:** Elodie Olivares, Stéphanie Badel-Berchoux, Christian Provot, Benoît Jaulhac, Gilles Prévost, Thierry Bernardi, François Jehl

**Affiliations:** ^1^Fédération de Médecine Translationnelle de Strasbourg, EA7290 Virulence Bactérienne Précoce, CHRU Strasbourg, Institut de Bactériologie, Université de Strasbourg Strasbourg, France; ^2^BioFilm Control SAS Saint-Beauzire, France

**Keywords:** *Pseudomonas aeruginosa*, cystic fibrosis, biofilms, aminoglycosides, Biofilm Ring Test, Antibiofilmograms, Crystal Violet, cell culture

## Abstract

Cystic fibrosis (CF) patients are predisposed to chronic colonization of the major airways by *Pseudomonas aeruginosa* biofilms. Pulmonary infections, involving sessile bacteria, are the main cause of morbidity and mortality. As the eradication of antibiotic-resistant biofilms remains impossible, one key objective for the treatment of lung infections is to delay the switch of *P. aeruginosa* to a sessile phenotype. Few tools are currently available in hospital laboratories to evaluate the susceptibility of adherent microorganisms to antimicrobials. In this study, we used the Biofilm Ring Test^®^, for the achievement of Antibiofilmograms^®^ on CF clinical isolates. In comparison to standard antibiograms, these procedures allow the investigation of antibiotic effects on the biofilm formation by bacteria. To confirm the inter-assay reproducibility, conventional Crystal Violet assays were performed. To mimic the pathologic reality of CF, we also used a model allowing the biofilm growth on CF-derived cells. Results obtained from these three different assays showed that amikacin and tobramycin, the two favored aminoglycosides in CF therapies, were able to prevent the early adhesion of *P. aeruginosa* isolates. This promising inhibitory effect of antimicrobials confirm that biofilm setting up is governed by adaptive responses and depends on environmental conditions, as opposite processes of biofilm induction by aminoglycosides were previously described in literature. Finally, Antibiofilmograms^®^, whose given results are in concordance with other *in vitro* antibiotic susceptibility testing, appear to be useful for the optimisation of CF therapies by the selection of antimicrobials able to delay chronic infection establishment.

## Introduction

Cystic fibrosis (CF) is the most common inherited genetic disorder in Caucasian populations (Lyczak et al., 2002). The pathology is bought by mutations in the gene for CF Transmembrane Conductance Regulator (CFTR) protein. Initially, CFTR is involved in production of sweat, digestive fluids and mucus. In the CF lungs, the chloride transport defect, caused by non-functional proteins, results in altered airway physiology, impairment of mucociliary clearance and production of thick mucus plugs. Consequently, CF individuals are predisposed to chronic pulmonary microbial infections, which lead to respiratory failure, the primary cause of patient death (Davies et al., 2007; Farrell et al., 2017).

*Pseudomonas aeruginosa* is the most frequent microorganism establishing chronic lung infections beyond infancy, in people with CF (Bhagirath et al., 2016). Indeed, this opportunistic pathogen possesses features that contribute to its adaptation and persistence in the CF lungs, despite the availability of aggressive use of antibiotics (Høiby et al., 2001). One of the main reason explaining the resistance of *P. aeruginosa* in the lower tract of CF patients is its ability to grow as a biofilm (Moreau-Marquis et al., 2008b).

This bacterial gathering, associated to the production of a protective polysaccharide matrix, renders microbial cells intrinsically more resistant to environmental agents and antimicrobials. The general principle of biofilm formation is the result of global regulatory modifications within the bacterium. The specific environment found in CF lungs seems to act as a stress signal governing the transition between acute (bacterial planktonic state) and chronic infections (bacterial sessile state) (Ciofu et al., 2015).

The aminoglycosides are an important class of antibiotics used to treat chronic bacterial infections, in particular lung infections. Among them, the superior activity of tobramycin (TOB) against *P. aeruginosa* makes it the preferred aminoglycoside for treatment of lung infections in CF. TOB by inhalation is often considered in first line for the eradication of early *P. aeruginosa* infections and for chronic therapies (Elborn et al., 2009). Amikacin (AMK) is also a good candidate as strains that are resistant to multiple other aminoglycosides tend to be susceptible to it. Its antimicrobial activity spectrum is the broadest of the group (Ehsan and Clancy, 2015).

The treatment of biofilm-involving infections is often poorly effective. Antibiotic susceptibility testing is traditionally performed on growing planktonic cells. Thus, antimicrobial selection does not take into account the common sessile growth of bacteria and antibiotic therapies cannot be effective on *in vivo* microbial biofilms (Ciofu et al., 2015). The development of clinical assays, evaluating the susceptibility of sessile microorganisms to antimicrobials, becomes essential particularly for the treatment of biofilm-associated infections as in CF (Moskowitz et al., 2011; Hurley et al., 2012).

The aim of the study was to evaluate the effects of AMK and TOB on the initiation of the biofilm formation by *P. aeruginosa* CF isolates, with the Biofilm Ring Test^®^ (BRT) system. This device allows the evaluation of the bacterial adhesion and the investigation of the antibiotic effect on the biofilm setting up (Antibiofilmogram^®^) (Olivares et al., 2016). Interestingly, prevention phenomena of bacterial adhesion were reported with various concentrations of AMK and TOB. Crystal Violet (CV) assay was also used as a comparison (Christensen et al., 1985; Stepanović et al., 2000). Finally, to attest that the delay of the biofilm formation initiation by the two aminoglycosides was not a specific response of the bacterial adhesion on abiotic surfaces, we also used a clinically more relevant cell model. The antibiotic effects were evaluated through a tissue culture-based system allowing the biofilm formation on CF airway epithelium (Anderson et al., 2008; Moreau-Marquis et al., 2010).

## Materials and Methods

### Bacterial Strains

Initially, 25 strains of *P. aeruginosa* were isolated from sputum of CF anonymized patients. They were collected from the CF foundation (Centre de Ressources et de Compétences de la Mucoviscidose) of the University Hospital of Strasbourg (France). A previous study allowed to classify them in three adhesion profiles according to their adherence ability and their speed of biofilm formation (Olivares et al., 2016). In this work, we focused on intermediate strains, namely the ones, which are able to progressively adhere within 6 h of incubation. In this way, two strains were selected for the following experiments: the strain #1 and the strain #24 of our clinical collection.

### Antibiotic Panel Selection

Two aminoglycosides, amikacin (AMK) and tobramycin (TOB) were used. Their Minimal Inhibitory Concentration (MIC) were determined on strains #1 and #24 by gradient diffusion strips (Etests, Biomérieux, France), following supplier’s indications (Marley et al., 1995).

Their effect on sessile cells was investigated at three concentrations: MIC, subMIC (half MIC) and a so-called “PK/PD” concentration (Jehl and Koebel, 2011). The latter is the concentration that allows the adequate PK/PD parameter relevant for aminoglycosides to reach their target. Indeed, the required concentration for optimal efficacy of aminoglycosides is 10 times the MIC. All the corresponding concentrations are summarized in the **Table 1**.

**Table 1:** Aminoglycoside concentrations tested on *P. aeruginosa* strains in Antibiofilmograms^®^, crystal violet and cell culture assays.

	(μg/mL)	Strain #1	Strain #24
AMK	**MIC**	**6**	**12**
	subMIC	3	6
	PK/PD	60	120
TOB	**MIC**	**0.75**	**0.5**
	subMIC	0.375	0.25
	PK/PD	7.5	5

*Antibiotic MIC is initially determined by *E*-tests. The effect of two others concentrations, on the bacterial adhesion, was investigated. The subMIC one corresponds to half of MIC and the PK/PD one reflects 10 times the MIC (*in vivo* efficacy of peak serum concentration).*

### Antibiofilmograms^®^

Antibiofilmogram^®^ consists in the characterisation of adhesion kinetics of bacteria in the presence of a given concentration of various antibiotics.

For BRT experiments, a fresh overnight culture of bacteria, grown on Brain Heart Infusion (BHI) agar plate, was used to inoculate 2 mL of sterile BHI medium. After determination of its OD_600nm_, Initial Bacterial Suspension (IBS) was prepared to an optical density adjusted to 1/250 (to reach approximately 10^6^ CFU/mL) in BHI medium, containing a final concentration of 10 μL/mL of the magnetic beads suspension (Pack BIOFILM Ring Test, Biofilm Control, Saint-Beauzire, France). 96-well polystyrene plates were inoculated with 200 μL/well of IBS after being loaded with 20 μL of antibiotic concentrations (**Table 1**). The U-bottom sterile microplates were directly provided by Biofilm Control. They are specifically no treated, enabling an optimal adhesion of microorganisms. One plate was prepared for each read incubation time (adhesion kinetics with antimicrobials were analyzed at 0, 2, 4, and 6 h).

Controls of spontaneous adherence of bacteria (without antibiotics) and migration of beads (medium without bacterial strains) were also prepared in each plate. After static incubation at 37°C, few drops of contrast liquid (inert opaque oil) were used to cover wells. Then, plates were magnetized 1 min with the block carrying 96 mini-magnets (Block test) and scanned with the BIOFILM Reader.

The adhesion strength of bacterial cultures with/without aminoglycosides was expressed as BioFilm Index (BFI), a score of beads aggregation. Briefly, a high BFI value signs bead mobility during magnetization step, namely no biofilm formation, while a low value shows a full bead immobilization due to the presence of sessile cells (Chavant et al., 2007).

Bacterial cultures supplemented with antibiotic concentrations were analyzed in six replicates to assess repeatability and precision of the Antibiofilmogram^®^. Two-way ANOVA analyses were applied to statistically compare resulting BFI data.

### Biofilm Detection with Crystal Violet (CV) Assay

For CV assays, the IBS were prepared in similar way as for BRT protocol and the microplates used were the ones provided by Biofilm Control. Sterile 96-well polystyrene plate was inoculated with 200 μL of IBS (10^6^ CFU/mL) in BHI medium and incubated for 6 h at 37°C. Twelve replicates for bacterial and antibiotic conditions were analyzed. After incubation, medium containing non-adherent cells was removed and wells were washed two times with 250 μL of sterile water. The plate was vigorously shaken during washing steps to remove all planktonic bacteria. The remaining adherent bacteria were stained with 250 μL of CV solution (0.1%) during 15 min. Then, the dye was removed and the wells washed three times with 250 μL of sterile water. From this step, adhesion of bacteria can be macroscopically visible in wells. Finally, the CV bound to the sessile cells was dissolved by 250 μL of acetic acid (30%) and the absorbance was measured at 540_nm_ by using the automated reader Micronaut Skan (Merlin Diagnostika GmbH, Bornheim, Germany). One-way ANOVA analyses were applied to statistically compare resulting OD values. We also defined a simple classification of our bacterial cultures according to their adherence ability, based on our experimental observations and measurements. First, the average OD values and standard deviations should be calculated for all tested conditions and negative controls. Second, the specific OD (OD_s_) should be established for bacterial cultures. It was defined as the mean OD of the condition subtracted by the mean OD of the negative control (BHI medium here). These final OD values were then compared to the mean OD of the negative control, as follows: OD_s_ ≤ OD_negative control_ + SD = non-adherent cells and OD_s_ > OD_negative control_ + SD = biofilm producer.

### Static Co-culture Biofilm Assay

To be closer to pathogenic reality of the CF, we adapted the tissue culture system of Anderson et al. (2008) to evaluate the adhesion of our clinical isolates on viable cells and the aminoglycoside effect on this biofilm formation (Moreau-Marquis et al., 2010). *P. aeruginosa* biofilms were grown on CF-derived human airway cells (CFBE41o^-^). This continuous cell line was generated by the transformation of bronchial epithelial cells collected in a CF patient, homozygous for the ΔF508 mutation of CFTR (Ehrhardt et al., 2006). Initially, epithelial cells were seeded at a concentration of 4.5 × 10^5^ cells/well in 24-well tissue culture plates or 7.5 × 10^5^ cells/well in 6-well tissue culture plates. They were maintained in Roswell Park Memorial Institute (RPMI) – 1640 medium (Dutscher SAS, Brumath, France) supplemented with 10% fetal bovine serum, 2 mM L-Glutamine, 10 U/mL penicillin and 10 μg/mL streptomycin. The cells were grown at 37°C and 5% CO_2_ until they form a confluent monolayer.

For bacterial inoculation, a fresh overnight BHI broth culture was prepared. The culture medium from CFBE41o^-^ cells was removed and they were washed three times with RPMI or phosphate-buffered saline (PBS). Afterward, they were inoculated with the bacterial suspension (^+^/_-_concentrations of aminoglycosides) in RPMI medium with L-Glutamine, at a Multiplicity Of Infection (MOI) of approximately 30:1, relative to the number of epithelial cells originally seeded. The co-cultures were incubated at 37°C and 5% CO_2_ for 6 h.

To determine the number of CFU in the biofilm, the medium containing planktonic bacteria was removed and cells were washed two times with PBS. Then, remaining co-cultures were treated with trypsin-EDTA to detach the cells and the associated adherent bacteria. The lysates were serially diluted and plated on BHI agar to perform a standard bacterial count. One-way ANOVA analyses were applied on resulting CFU/mL data for the statistical study of results.

Alternatively, calcofluor staining can also be achieved to observe biofilm structures developed on cell monolayer. Biofilm staining was assessed by fluorescence microscopic examination (Olympus System Microscope Model BX60 with immersion objective ×40), after incubation of washed co-cultures with calcofluor 1% for 30 min.

## Results

### Evaluation of Aminoglycoside Effect on Early Bacterial Adhesion by Antibiofilmograms^®^

**FIGURE 1:**
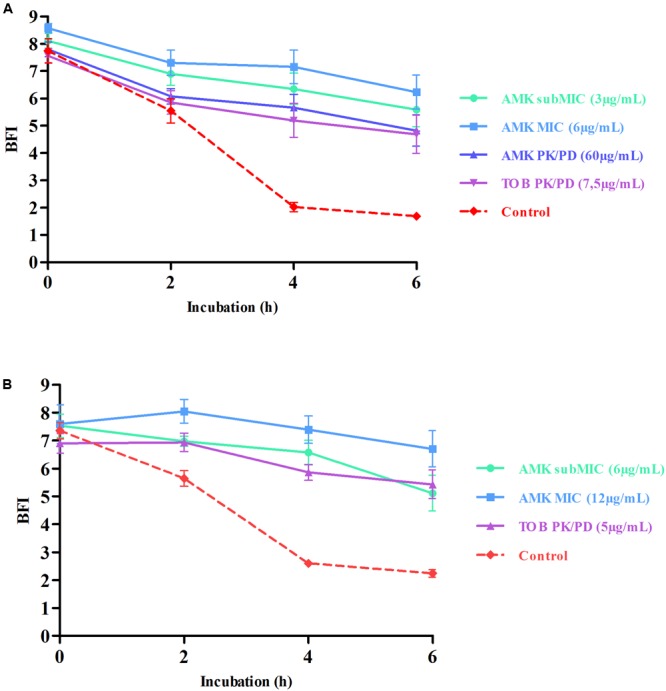
Prevention of bacterial adhesion by aminoglycosides highlighted by Antibiofilmogram^®^ results of strains #1 **(A)** and #24 **(B)**. BioFilm Index (BFI) are expressed as a function of incubation time. This index represents a score of beads aggregation. More precisely, a high BFI value signs no biofilm formation, while a low value shows the presence of sessile cells. Red dotted lines represent control strains (without antibiotic) and continuous curves symbolize adhesion kinetics of bacteria in contact with different concentrations of aminoglycosides for 6 h. The standard deviations represent the mean of six BFI measurements. Statistical analyses have been performed through two-way ANOVA testing and showed that curves with antibiotics are significantly different from the control one at 4 and 6 h of incubation (*p* < 0.0001). The bead immobilization by bacteria is delayed by antimicrobials and reflects the prevention of the bacterial adhesion.

The realization of Antibiofilmograms^®^, through the BRT device, allows the ability evaluation of antimicrobials to prevent the growth of bacteria in a sessile state, conducting to the formation of a biofilm. Results of assays testing aminoglycoside effects on strains #1 and #24 are presented in **Figure 1**. The control curves confirmed the spontaneous progressive adhesion of bacteria. For the two strains, the gradual immobilization of magnetic beads by the biofilm formation initiation is reflected by the decrease of the BFI values during the time experiment (a BFI value near of 2 represents a complete sessile state). Conversely, the use of AMK and TOB at different concentrations slowed down the adhesion of bacteria, as shown by higher BFI values, starting from 4 to 6 h of incubation. Result analyses by two-way ANOVA testing validated a statistically significant difference between points of antibiotic curves and the control ones at 4 and 6 h of incubation (*p* < 0.0001).

Effects of antimicrobials belonging to antibiotic families of β-lactams and fluoroquinolones were also evaluated with the BRT device. Results showed that none of them prevented or reduced the progressive adherence of bacteria (data not shown). The only significant effects obtained with our two clinical strains were the inhibitory process of bacterial adhesion by aminoglycosides.

### Categorical Concordance of Antibiofilmogram^®^ Results with CV Staining

**FIGURE 2:**
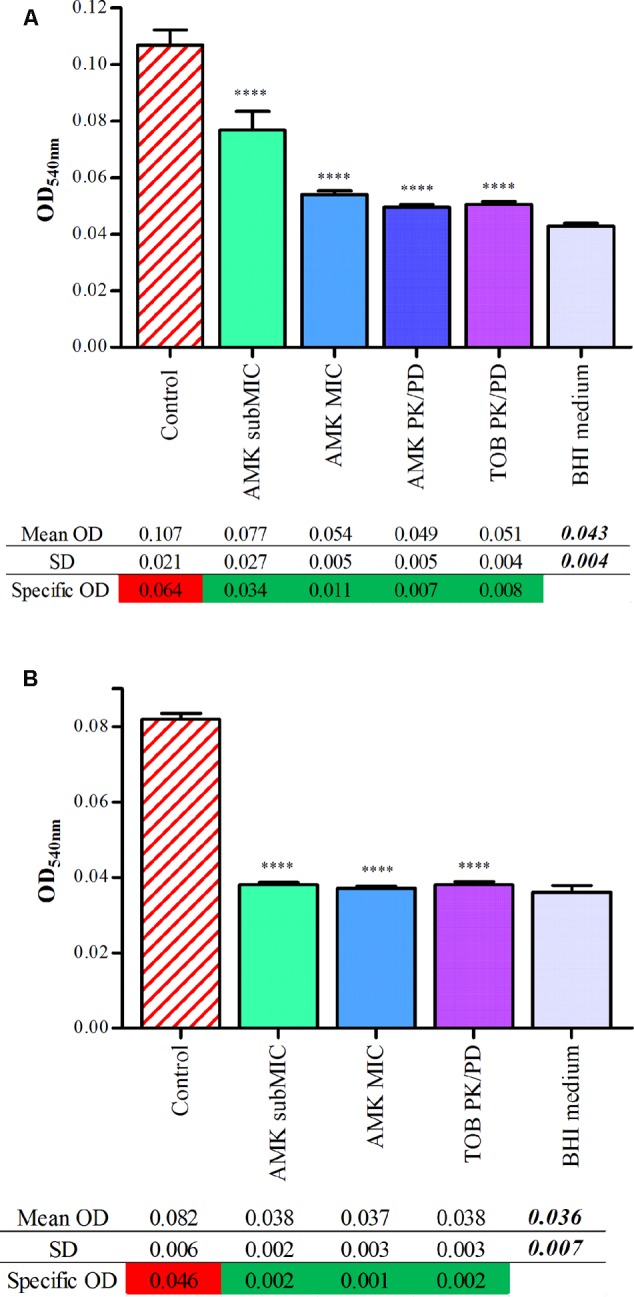
Quantitative analysis of adherence of strains #1 **(A)** and #24 **(B)** with aminoglycosides by CV staining. Graphs represent adherence quantification by CV assay. Each bar associated to standard deviation reflects the mean OD_540nm_ of *P. aeruginosa* ^+^/_-_ aminoglycoside concentrations obtained from 16 replicates for each condition. Statistical analyses have been performed through one-way ANOVA testing for the comparison of each antibiotic concentration with control strains (*p* < 0.0001). Starting from their OD_s_ value (mean OD of the analyzed condition subtracted by the mean OD of the negative control), bacterial cultures were classified as follows: OD_s_ ≤ OD_negative control_ + SD = non-adherent cells (green highlighting); OD_s_ > OD_negative control_ + SD = biofilm producer (red highlighting).

The spontaneous adhesion by clinical isolates and its prevention by aminoglycosides were further assessed by the CV assay. The average light absorbance of biofilms formed by *P. aeruginosa* strains, associated with AMK and TOB, are represented in **Figure 2**. Raw data of OD_540nm_, reflecting the CV bound by sessile cells, showed that strains #1 and #24 adhere spontaneously after 6 h of incubation, as for controls of Antibiofilmograms^®^. A drop of OD values, allowing their reclassification as non-adherent cells, was observed for cultures of bacteria with antibiotics. The statistical analysis of the OD_540nm_ means confirmed a significant difference of CV bound between control strains and cultures with AMK and TOB (*p* < 0.0001). These results attest that aminoglycosides, at the tested concentrations, limit the adhesion of bacteria.

A general agreement was recorded between CV and Antibiofilmogram^®^ assays concerning the ability of aminoglycosides to delay the initiation of the biofilm formation by clinical *P. aeruginosa* strains.

### Bacterial Adhesion Prevention and Biomass Reduction on CFBE41o^-^ Cells

**FIGURE 3:**
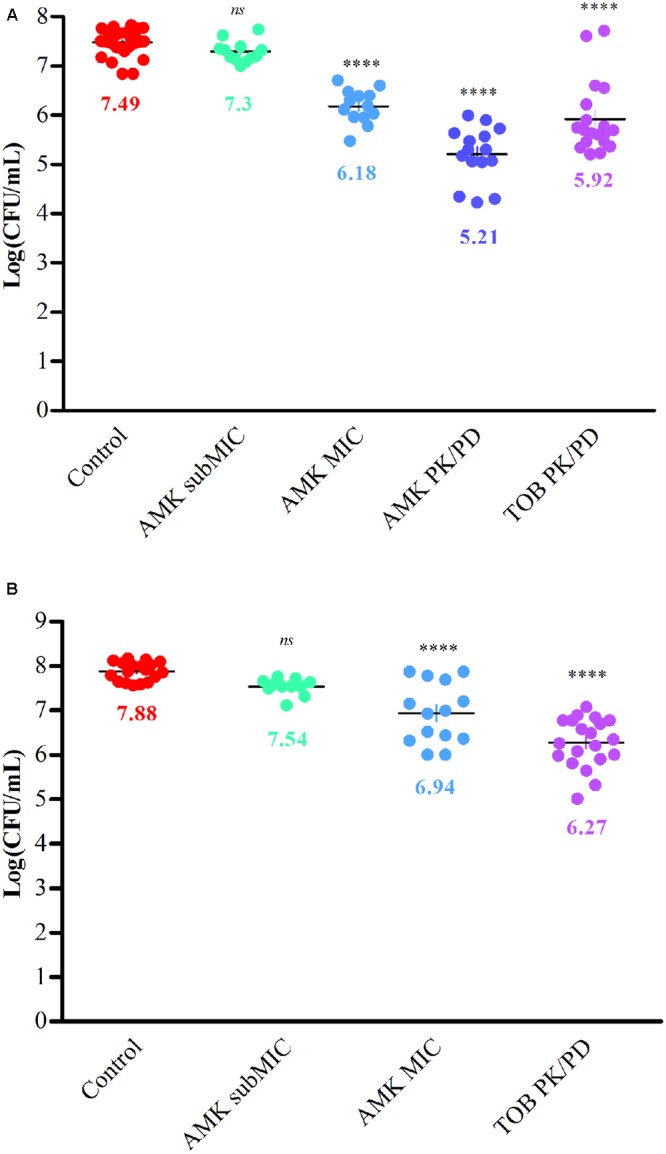
Reduction of bacterial biomasses adhered to CFBE41o^-^ cells by aminoglycosides for strains #1 **(A)** and #24 **(B)**. Scatter plots represent counts of *P. aeruginosa* colonies ^+^/_-_ various concentrations of aminoglycosides after a 6h-culture with CFBE41o^-^ cells. Bacterial numerations are expressed as log CFU/mL. The spontaneous adhesion of control strains to the cell monolayer is confirmed (>7log). A decrease of the adhered bacterial biomass was observed with aminoglycosides. Data reflect three independent co-culture experiments and statistical analyses have been performed through one-way ANOVA testing for the comparison of each antibiotic concentration with control strains (*p* < 0.0001).

The inhibitory effect of AMK and TOB on bacterial adhesion was investigated on CF-derived cells. The bacterial numerations, reflecting the number of adherent bacteria bound to the cell layer after 6 h of incubation, are expressed in **Figure 3**. Similar bacterial behaviors to those previously observed with BRT and CV assays were confirmed for strains #1 and #24. First, bacteria were spontaneously able to adhere to the cell monolayer. The initial inocula for the two control strains reflect more than 3 × 10^7^ CFU/mL for strain #1 and 7.5 × 10^7^ CFU/mL for strain #24. For bacterial cultures with antibiotics, a decrease of adherent biomasses was observed. The drop of bacterial counts can reach more than one log for the highest antibiotic concentrations. Compared with spontaneous adhesion of control strains, a statistically significant reduction of the biomass was observed with concentrations of aminoglycosides equal to MIC and PK/PD values. No effect of the subMIC concentration of AMK was observed on bacterial adherence with the strain #1.

**FIGURE 4:**
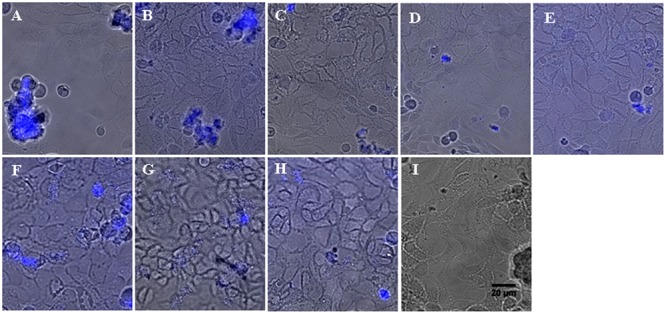
Representative images of *P. aeruginosa* microcolonies grown on confluent human CF-derived cells after 6 h of incubation, in absence or presence of aminoglycosides. Spontaneous formation of microcolonies by strains #1 **(A)** and #24 **(F)** was assessed on CFBE41o^-^ cells by epifluorescence microscopy. Blue fluorescence resulted from the staining of the bacterial polysaccharide matrix with calcofluor. Image is an overlay of the phase contrast channel and the fluorescence channel. The observation plane was focused on the cell monolayer, explaining why the bacterial 3D-structures located above appeared somewhat blurred. Addition of AMK at subMIC (**B** for strain #1 and **G** for #24), MIC (**C** for #1 and **H** for #24) and PK/PD concentrations (**D** for #1) prevented the formation of bacterial clusters. In the same way, the PK/PD concentration of TOB inhibited the bacterial adhesion on epithelial cells (**E** for #1 and **I** for #24). Scale bar, 20 μm.

The staining of cultures with calcofluor was simultaneously performed to verify that the decrease of bacterial adhesion observed with aminoglycosides is correlated to a reduction of microcolony establishment. Briefly, calcofluor is a fluorescent stain able to bind structures containing cellulose and chitin and more generally polysaccharides. In biofilm studies, its use is appropriate for the slime detection. Fluorescence microscopy revealed clusters of *P. aeruginosa* cells scattered across the epithelial cell monolayer for the control strains #1 and #24 (**Figures 4A,F**, respectively). These clusters appeared to be attached to the cells and exhibited a 3D-structure. Moreover, in comparison of the flat layer of epithelial cells, these bacterial clusters were strongly stained with calcofluor, suggesting the presence of a polysaccharide matrix. These morphological observations showed that the strains #1 and #24 were able to initiate a biofilm formation on the CFBE41o^-^ cells. These results confirmed once again the spontaneous sessile state of bacteria, observed with BRT, CV and bacterial enumeration experiments. As for CFU counts, a decrease of stained-biofilm structures was observed for cultures with antibiotics (**Figures 4B–E** for strain #1, **Figures 4G–I** for strain #24). The average number and the size of observed clusters decreased significantly with the highest concentrations of AMK and TOB. For the PK/PD ones, the clusters were even flat and seemed to be an early gathering of bacteria (**Figure 4E** for strain #1, **Figure 4I** for strain #24). Fluorescence observations confirmed the prevention of the early bacterial adhesion by aminoglycosides and were an additional proof of agreement of previous results obtained with *in vitro* CV and Antibiofilmogram^®^ assays.

## Discussion

Pulmonary infections are the main cause of morbidity and mortality in patients with CF. The lung colonization is essentially dominated by *P. aeruginosa*, which grows in a sessile state. This biofilm formation leads to a chronic inflammation in the airways and owing to its antimicrobial resistance, makes eradication of the infection impossible (Moreau-Marquis et al., 2008b; Bhagirath et al., 2016). As common antibiotic susceptibility testing do not indicate the tolerance of biofilm-growing bacteria, the development of laboratory assays, taking into account the *in vivo* sessile state of microorganisms is essential. This kind of diagnostic tools could be easily incorporated in routine clinical laboratory testing to provide complementary data for the antibiotic selection.

Antibiofilmograms^®^ allowed the study of adhesion kinetics of sessile bacteria and the evaluation of their susceptibility to antimicrobials through the BRT device. In our study, realization of these assays showed that our *P. aeruginosa* clinical strains are susceptible to various concentrations of AMK and TOB, two molecules considered as antimicrobials of first choice for treatment of pulmonary infections in CF. Both aminoglycosides delayed the initiation of the biofilm formation by bacteria.

These results were confirmed by the conventional CV assay. Finally, the inhibitory effect of aminoglycosides was confirmed with CF-derived cells. Only one discriminating result was noticed between the three applied methodologies: the inhibitory potential of the subMIC concentration of AMK on the bacterial adhesion prevention of the strain #1, when compared to the cell model which detects no antimicrobial effect. Effects of antimicrobials belonging to the families of β-lactams and fluoroquinolones were also tested with BRT, CV and cell co-culture assays. Clearly, these antibiotics did not disturb the spontaneous adhesion ability of bacteria (data not shown).

In opposition to our results, previous works have described the ability of subinhibitory concentrations of aminoglycosides, particularly tobramycin, to induce the biofilm formation in *P. aeruginosa* (Hoffman, 2005; Elliott et al., 2010). This deleterious effect of antimicrobials is explained as being an adaptive response of microorganisms to antibiotic exposure (Kaplan, 2011). Indeed, antimicrobials were presented as signal agents for the homeostasis regulation of microbial communities. As bacteria coevoluate in the environment under constant weak concentrations of antibiotics, they have developed a protective response by the formation of resistant biofilms (Linares et al., 2006). In our study, the subMIC concentration of TOB did not show deleterious effect on the bacterial adhesion (data not shown). These opposite effects of antimicrobials on microbial adhesion could be explained by the mucoid phenotype of our bacterial isolates. We intentionally used mucoid strains for co-cultures to allow the staining and the observation in fluorescence of the biofilm matrix. Mucoid isolates have been described as able to maintain their antibiotic susceptibility in comparison of non-mucoid strains, which rapidly develop resistance by their hypermutability (Oliver et al., 2000; Ciofu et al., 2001). Kaplan (2011) also introduce the notion of hormetic responses by antimicrobials. In fact, the bacterial biofilm formation exhibits a biphasic response to antimicrobial exposure. In this sense, antibiotics can act as antagonist or agonist according to the concentration tested (Kaplan, 2011). Finally, it seems established that the biofilm formation is mainly governed by adaptive responses. Indeed, transcriptomic analyses have failed to identify specific biofilm regulons suggesting that its formation is dependent on the environmental conditions (Whiteley et al., 2001; Ciofu et al., 2015).

Antibiofilmograms^®^ revealed that AMK and TOB decrease the bacterial adhesion as paramagnetic beads stay free-moving for these conditions. This tool allows rapid screen of antibiotics and does not require fixation or staining procedures. Its use could be appropriate to precise CF treatments as it is specifically intended for analyze of the early stages of biofilm formation. The major limitation of the conventional CV microtiter plate assay is the bias of the bacterial sessile biomass estimation due to the repetitive washing steps. It is even advisable to add a fixation step with ethanol or heat, just before the dye staining step, to prevent the detachment of adhering microorganisms (Stepanović et al., 2007). Even without this binding step, which usually enhances reproducibility of the assay, we found reproducible results confirming the preventive effect of aminoglycosides. Besides, we introduced our own classification of bacterial cultures according to adhesion potential of microorganisms. Based on our experimental observations, we decided to directly compare the final mean OD value of conditions to the upper OD of the negative control (mean OD + SD). This methodology differs from the one originally described by Stepanović et al. (2007), which defined a cut off OD to compare to final OD of the tested conditions. In terms of experiments carried out on only few hours of incubation, as for our CV assays, this method of classification undervalues the potential of bacteria to adhere to a support. Finally, we adapted a static tissue co-culture system to allow the adhesion, of our clinical isolates, on human CF-derived airway cells. Our clinical strains were able to bind to the cell layer in forming microcolonies and more precisely, 3D-structures. The latter were characterized by the presence of polysaccharide matrix, revealed by a calcofluor staining. In this co-culture model, AMK and TOB showed the ability to decrease the bacterial viability and the formation of a protective matrix. Previous studies have also evaluated the potential toxicity of antimicrobials on the airway cells. No evidence of cytotoxicity was observed for tobramycin treatment of *P. aeruginosa* biofilms on CFBE cells (until 1000 mg/L) (Anderson et al., 2008; Moreau-Marquis et al., 2009; Yu et al., 2012). Although this kind of *in vitro* cell model cannot be standardized in diagnostic laboratories, it is closer to the *in vivo* pathologic reality than microtiter plate assays. Even studies have suggested that in CF airways, the biofilm formation by *P. aeruginosa* is initiated within the thick mucus, in the early stages of lung colonization, bacteria can directly interact with epithelial cells (Anderson et al., 2008). In that sense, the cell system allows the evaluation of biofilm formation on another support than polystyrene microplates and with a different culture medium. It is well conceivable that according to the adhesion surface, the ability of biofilm formation varies. With tissue culture systems, it has already been observed differences of bacterial responses to antimicrobials according to the epithelial cell lines and even according to the CFTR status of cells, in the case of CF studies (Moreau-Marquis et al., 2008a). In the same manner, with *in vitro* assays, the medium composition may have a strong impact on the biofilm setting up ability of bacteria. To explore this issue, we have preliminary tested the adhesion ability of our strains, by microscopic observations, in BHI and RPMI + L-Glutamine media. We found a spontaneous adhesion of bacteria to microplates for the two culture media but in a much higher proportion for the RPMI one (data not shown). This medium seems to significantly stimulate the initial adherence of *P. aeruginosa*. This difference could be due to the poorer nutrients composition in RPMI than in BHI. The nutrient stress is a well-known parameter increasing the biofilm formation. Nevertheless, despite this adhesion stimulation, aminoglycosides could delay the bacterial adhesion in the co-culture experiments.

Taken together, results obtained by three inherently different assays confirm the ability of aminoglycosides to prevent the early adhesion of CF clinical *P. aeruginosa* strains. Currently, inhalations with TOB are predominantly favored for the maintenance therapy of chronically *P. aeruginosa* infected patients. Topical administration of antibiotics allows to reach high concentrations, in contrast with systemic treatments. In conclusion, the BRT device provides complementary information about antimicrobial effects on sessile bacterial cells. These data, being more relevant compared to a classical antibiogram, insist on its utility in routine hospital practice. Its robustness was confirmed by conventional and cell assays. Finally, we attested that aminoglycoside family, which is promptly administered to chronic infected CF patients, has a beneficial impact in delaying the initiation of the biofilm setting up by *P. aeruginosa* strains.

## Author Contributions

FJ and EO designed the study. EO performed the experiments, analyzed the data and wrote the manuscript. All authors read and approved the final manuscript.

## Conflict of Interest Statement

EO, SB-B, CP and TB declare a conflict of interest: SB-B, CP and TB are members of BioFilm Control SAS, Saint-Beauzire, France. A CIFRE fellowship (Ph.D. student) from BioFilm Control, for EO, is gratefully acknowledged. The other authors declare that the research was conducted in the absence of any commercial or financial relationships that could be construed as a potential conflict of interest.
